# Complete chloroplast genome sequence of *Oxytropis glabra* (Leguminosae)

**DOI:** 10.1080/23802359.2021.1914228

**Published:** 2021-07-26

**Authors:** Shuo Liu, Ya-Rui Li Wei, Wei Si, Wen-Rui Qu, Tian-Ge Yang, Zhi-Hua Wu, Pei-Pei Jiao

**Affiliations:** a Hubei Provincial Key Laboratory for Protection and Application of Special Plant Germplasm in Wuling Area of China, College of Life Sciences, South-Central University for Nationalities, Wuhan, China; b Security Department of Tarim University, Alar, China; c Xinjiang Production and Construction Corps Key Laboratory of Protection and Utilization of Biological Resources in Tarim Basin, College of Life Science, Tarim University, Alar, China; d College of Life Science and Technology, Huazhong Agricultural University, Wuhan, China

**Keywords:** *Oxytropis glabra*, chloroplast genome, evolution

## Abstract

*Oxytropis glabra* DC. is a perennial poisonous plant to livestock belonging to the genus *Oxytropis,* Leguminosae, mainly distributed in Northwestern China. As a poisonous grass, this species protects plant diversity in degraded grasslands by sheltering adjacent plants. In this study, the complete chloroplast genome with a total size of 122,094 bp was reported. Our annotations showed that the chloroplast genome contains 109 genes, including 76 protein-coding genes, 29 tRNA genes, and four rRNA genes. This work presents complete chloroplast genome information, which will be valuable for studying the evolution and genetic diversity of *O. glabra.*

*Oxytropis glabra* DC. (Leguminosae) is a perennial poisonous plant to livestock belonging to Leguminosae, mainly distributed in Northwestern China. As a common poisonous grass, *O. glabra* protects plant diversity in degraded grasslands by sheltering adjacent plants. In addition, some species of *Oxytropis* were reported to have high flavonoids, such as *Oxytropis falcata* Bunge. The whole herb of *Oxytropis falcata* has a variety of pharmacological activities, including anti-inflammatory and antioxidant drugs (Wang et al. 2010; Yang et al. 2010). In order to reveal the phylogeny of *O. glabra*, we sequenced the genome, assembled and annotated the complete chloroplast genome.

In this study, the materials of *O. glabra* were collected from Akqi County, Xinjiang province of China (78.675°E, 41.006°N, 1837 m above sea level). The voucher specimen (TD-00572, *Oxytropis glabra* DC.) was stored in the herbarium of Tarim University. The total genomic DNA from leaves was extracted using CTAB method (Doyle and Doyle 1987) and sequenced using the Illumina NovaSeq platform at Majorbio Company (Shanghai, China). First, the clean data were quality-controlled by using FastQC v0.11.9 (http://www.bioinformatics.babraham.ac.uk/projects/fastqc/). The whole chloroplast genome was assembled using GetOrganelle v1.7.3 (Jin et al. 2020). Then, to check the accuracy of assembly results, the slimmed assembly graph and selected target assembly graph can be visualized by Bandage v0.8.1 (https://github.com/rrwick/Bandage/releases/tag/v0.8.1) to assess the completeness of the final graph. Finally, the final assembly result is obtained. Gene annotation was performed using CPGAVAS2 (http://47.96.249.172:16019/analyzer/annotate) (Shi et al. 2019) and PGA (https://github.com/quxiaojian/PGA) (Qu et al. 2019). The differential annotations of protein-coding sequences were confirmed using BLASTx in NCBI. We obtained a complete chloroplast genome of 122,094 bp (MW349014) that lost an IR region and included average GC content of 34.3%. Most chloroplast genome are characterized by a quadripartite structure, which include two copies of an inverted repeat (IR) separating the large (LSC) and small (SSC) single copy regions. But some tribes among legumes have a common phenomenon that losing one copy of the IR in the chloroplast genome, such as Carmichaelieae, Cicereae, Hedysareae, Trifolieae, Fabeae, Galegeae, and three genera of Millettieae (Palmer and Thompson 1982; Lavin et al. 1990; Liston 1995; Jansen et al. 2008). The phenomenon may be a special feature of legumes in the evolutionary process. In this study, we showed that the complete chloroplast genomes encoded 109 functional genes, containing 76 protein-coding genes, 29 tRNA genes, and four rRNA genes.

To reveal the phylogenetic relationship of *O. glabra* within Leguminosae, additional 14 species from Leguminosae were selected to study. With the *Polygala japonica* and *Polygala tenuifolia* as the outgroups, the phylogenetic trees were built from the 76 protein-coding gene matrixes by maximum-likelihood (ML) and Bayesian inference (BI) (Figure 1). The ML tree was generated using IQ-TREE v2.1.2 (Nguyen et al. 2015) based on the best model of TVM + F+R2 and 1000 bootstrap replicates, and BI analysis was performed in MrBayes v3.2.7 (Ronquist et al. 2012). This result showed that the analyzed *O. glabra* was clustered with *O. splendens* and *O. arctobia*, all of which showed closer to the species of *Lessertia frutescens* and *Sphaerophysa salsula*.

**Figure 1.**
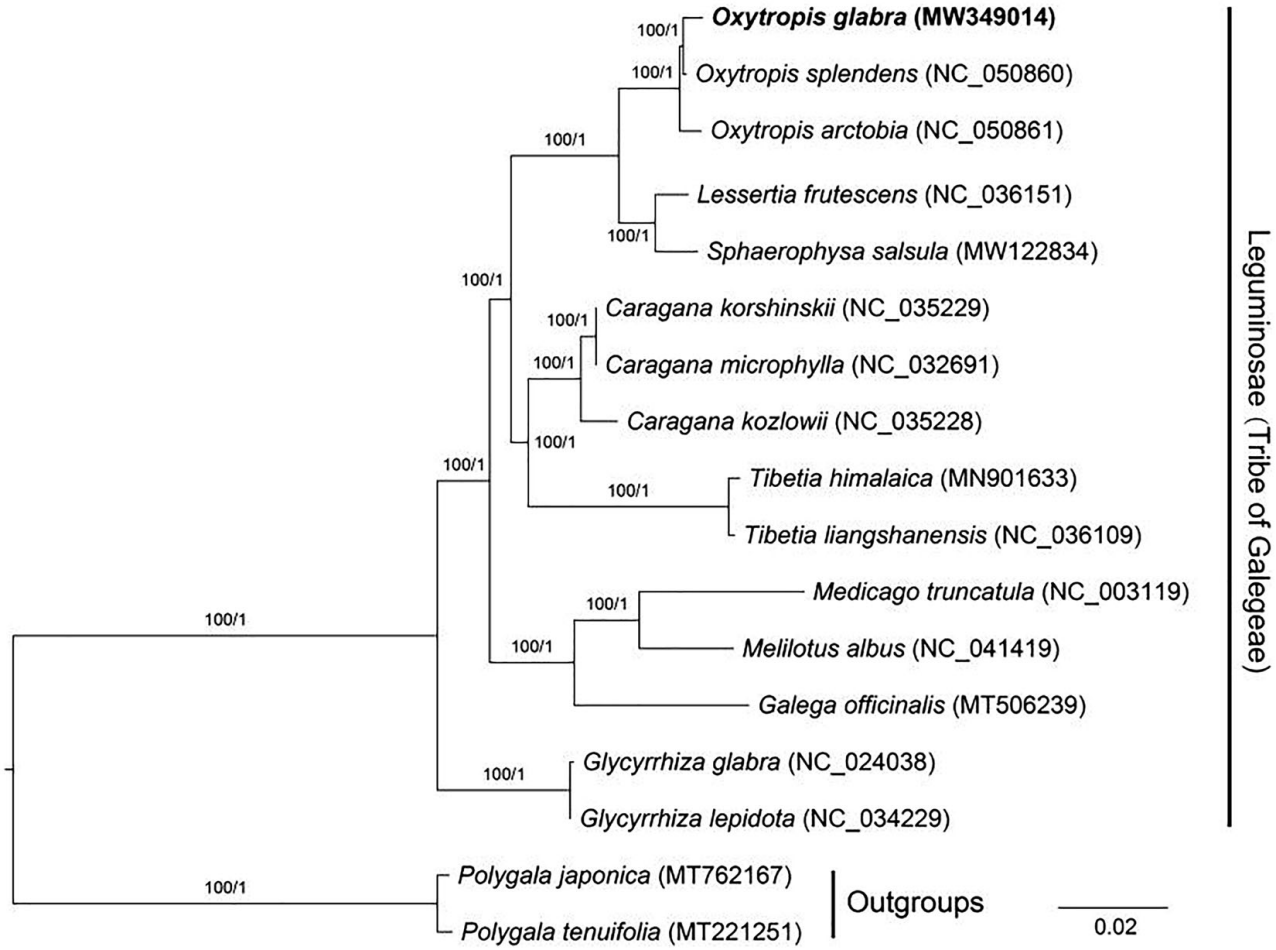
Phylogenetic tree reconstructed by maximum-likelihood (ML) and Bayesian inference (BI) analysis based on the 76 chloroplast protein-coding genes of these 17 species.

## Data Availability

The genome sequence data that support the findings of this study are openly available in GenBank of NCBI at [https://www.ncbi.nlm.nih.gov] (https://www.ncbi.nlm.nih.gov/) under the accession no. MW349014. The associated “BioProject”, “SRA”, and “Bio-Sample” numbers are PRJNA686236, SRR13275092, and SAMN17109568 respectively.
